# MicroRNA-296: a promising target in the pathogenesis of atherosclerosis?

**DOI:** 10.1186/s10020-018-0012-y

**Published:** 2018-03-26

**Authors:** Heng Li, Xin-Ping Ouyang, Ting Jiang, Xi-Long Zheng, Ping-Ping He, Guo-Jun Zhao

**Affiliations:** 1 The Clinic Medical College, Guilin Medical University, No. 1 Zhiyuan Road, Guilin, Guangxi 541100 China; 2 Hunan Province Cooperative innovation Center for Molecular Target New Drug Study, 28 West Changsheng Road, Hengyang, Hunan 421001 China; 3 Department of Physiology, The Neuroscience Institute, Medical College, University of South China, Hengyang, Hunan 421001 China; 4 Department of Practice educational, Office of Academic Affairs, Guilin Medical University, Guilin, 541100 China; 5 Department of Biochemistry and Molecular Biology, The Libin Cardiovascular Institute of Alberta, The University of Calgary, Health Sciences Center, 3330 Hospital Dr. NW, Calgary, AB T2N 4N1 Canada; 6 Nursing School, University of South China, Hengyang, Hunan 421001 China; 7 Department of Histology and Embryology, Guilin Medical University, Guilin, Guangxi 541004 China; 8 Key Laboratory of Molecular Targets & Clinical Pharmacology, School of Pharmaceutical Sciences, Guangzhou Medical University, Guangzhou, Guangdong 511436 China

**Keywords:** miR-296, Angiogenesis, Inflammation, Atherosclerosis

## Abstract

Atherosclerosis has been recognized as an inflammatory disease involving the vascular wall. MicroRNAs are a group of small noncoding RNAs to regulate gene expression at the transcriptional level through mRNA degradation or translation repression. Recent studies suggest that miR-296 may play crucial roles in the regulation of angiogenesis, inflammatory response, cholesterol metabolism, hypertension, cellular proliferation and apoptosis. In this review, we primarily discussed the molecular targets of miR-296 involved in the development of atherosclerosis, which may provide a basis for future investigation and a better understanding of the biological functions of miR-296 in atherosclerosis.

## Introduction

The atherosclerotic cardiovascular disease is the main cause of death in developing and industrialized countries (Barquera et al. 2015). The pathogenesis and development of atherosclerosis are mechanistically associated with many pathophysiological factors, such as angiogenesis, inflammatory response, cholesterol metabolism, hypertension, cellular proliferation and apoptosis (Buckley and Ramji 2015; Camaré et al. 2017).

MicroRNAs (miRNAs) are endogenous small (20–22 nucleotides long) non-coding RNAs that can post-transcriptionally regulate metazoan gene expression. The underlying mechanisms involve either induction of mRNA degradation or direct inhibition of protein translation (Andreou et al. 2015; Selbach et al. 2008). MiRNA regulation is characterized by its active participation in and strict control of the negative feedback loop, which confers significant influences on a biological pathway (Ebert and Sharp 2012; Laffont and Rayner 2017). To date, accumulating experimental evidence indicates that miRNAs, which are involved in every stage of biological processes and the changes of their expression and/or function have been associated with the initiation and progression of atherosclerosis (Giral et al. 2016; Laffont and Rayner 2017).

**Fig. 1:**
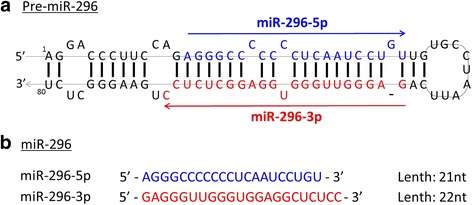
The structure of the 80 nt pre-miR-296 (pre-miR-296) precursor and mature miR-296. **a** *Homo sapiens* stem-loop structure of pre-miR-296. Two different mature miRNAs are produced from this precursor: miR-296-5p (sequence in blue) and miR-296-3p (sequence in red). The orientations (5′ to 3′) are indicated by the direction of the arrows. **b** Mature sequence of hsa-miR296-5p and hsa-miR296-3p

MiR-296 is a family of microRNA precursors found in mammals, including humans, which is located in the chromosome 20q13.32 genomic locus. The miR-296 precursor generally gives rise to mature miR-296. It is called miRNA-296-5p if derived from the 5′ arm, and miRNA-296-3p if derived from the 3′ arm (Fig. 1). In most, but not all, tissues, the expression of miR-296-5p is higher than miR-296-3p (Robson et al. 2012). There has been a close association between miR-296 expression and cardiovascular diseases. MiR-296-5p is upregulated in patients with hypertrophic cardiomyopathy (Fang et al. 2015). Wei et al. also found that miR-296-5p is one of the five miRNAs that were significantly elevated in early plaque formation and did not change significantly in late plaques in apoE^−/−^ mice fed high-cholesterol diet, suggesting that miR-296-5p may be a risk factor for atherosclerosis (Wei et al. 2013).

Studies have shown that miR-296 can be expressed in endothelial cells, macrophages and vascular smooth muscle cells, which are the major cell types involved in the development of atherosclerotic plaque (Feng et al. 2015; Meng et al. 2014; Reddy et al. 2016; Zheng et al. 2015). The atheromatous lesions showed distinct VEGF positivity of activated endothelial cells (Inoue et al. 1998) and VEGF alone is capable of increasing miR-296, (Würdinger et al. 2008) suggesting miR-296 may be expressed in endothelial cells in atherosclerotic lesions. Latent tuberculosis infection (LTBI) is associated with persistent chronic inflammation and has pro-atherogenic effect in human vessels (Huaman et al. 2015). Identification of LTBI infection-related miRNAs in human macrophages showed that miR-296-5p was significantly increased than the uninfected cells (Meng et al. 2014; Zheng et al. 2015). Type 1 and type 2 diabetes are associated with an enhanced inflammatory state and that inflammatory cells contribute to atherosclerotic lesion initiation and lesion disruption (Kanter et al. 2008). Reddy and colleagues found that miR-296 was upregulated in db/db VSMC compare to db/+ VSMC via small RNA-sequencing (Reddy et al. 2016). However, the function of miR-296, especially its role in atherosclerosis, has not been extensively studied.

MiR-296 has many bioinformatics targets that have been predicted and biologically validated in human and mouse (Table 1), suggesting that miR-296 has a wide range of potential biological functions. In this overview, we summarized the current knowledge regarding the potential roles of miR-296 in atherosclerosis (Table 2) and tried to provide a basis for future investigation and expand our insights into miR-296 functions in atherosclerosis.

**Table 1:** Bioinformatics targets of miR-296 that were predicted and biologically validated in human and mouse

miRNA Name	Target gene	Gene Description	Bioinformatics prediction tool	Biological validation
hsa-miR-296-5p	HMGA1	high mobility group AT-hook 1	miRDB, TargetScan	Yes
hsa-miR-296-5p	NUMBL	numb homolog (Drosophila)-like	miRDB, TargetScan	Yes
hsa-miR-296-5p	BOK	BCL2-related ovarian killer	miRDB	Yes
hsa-miR-296-3p	CX3CR1	chemokine (C-X3-C motif) receptor 1	miRDB	Yes
mmu-miR-296-5p	Pin1	protein (peptidyl-prolyl cis/trans isomerase) NIMA-interacting 1	miRDB	Yes
hsa-miR-296-3p	PTEN	phosphatase and tensin homologue	TargetScan	Yes
hsa-miR-296-5p	NGFR	nerve growth factor receptor	TargetScan	Yes
hsa-miR-296-5p	FGFR1	fibroblast growth factor receptor 1	TargetScan	Yes
hsa-miR-296-3p	SOCS6	suppressor of cytokine signaling 6	miRDB, TargetScan	No
hsa-miR-296-5p	RNF44	ring finger protein 44	miRDB, TargetScan	No
hsa-miR-296-5p	EPN1	epsin 1	miRDB, TargetScan	No
hsa-miR-296-3p	ZBTB20	zinc finger and BTB domain containing 20	miRDB, TargetScan	No
mmu-miR-296-3p	ZBTB20	zinc finger and BTB domain containing 20	miRDB, TargetScan	No
mmu-miR-296-3p	STRN3	striatin, calmodulin binding protein 3	miRDB, TargetScan	No
hsa-miR-296-5p	LYPLA2	lysophospholipase 2	miRDB, TargetScan	No
mmu-miR-296-5p	DYRK1B	dual-specificity tyrosine-(Y)-phosphorylation regulated kinase 1b	miRDB, TargetScan	No
mmu-miR-296-5p	LYPLA2	lysophospholipase 2	miRDB, TargetScan	No
mmu-miR-296-5p	Srf	serum response factor	miRDB, TargetScan	No
mmu-miR-296-5p	SRRM3	serine/arginine repetitive matrix 3	miRDB, TargetScan	No
hsa-miR-296-3p	LMLN	leishmanolysin-like (metallopeptidase M8 family)	miRDB	No
hsa-miR-296-3p	KLHL23	kelch-like family member 23	miRDB	No
hsa-miR-296-3p	ZNF117	zinc finger protein 117	miRDB	No
hsa-miR-296-3p	CADPS	Ca^++^-dependent secretion activator	miRDB	No
hsa-miR-296-3p	NTF3	neurotrophin 3	miRDB	No
-hsa-miR-296-5p	TEAD3	TEA domain family member 3	TargetScan	No
hsa-miR-296-5p	BBC3	BCL2 binding component 3	TargetScan	No
hsa-miR-296-5p	ISCA2	iron-sulfur cluster assembly 2	TargetScan	No
hsa-miR-296-5p	RIMS4	regulating synaptic membrane exocytosis 4	TargetScan	No
mmu-miR-296-3p	SOCS6	suppressor of cytokine signaling 6	TargetScan	No

**Table 2:** The target genes of miR-296 and its roles in atherosclerosis

Target gene	Pathways	Function	Potential effect in As	Ref.
HGS	PDGFR-β,VEGFR2	pro-angiogenesis	↑	(Würdinger et al. 2008)
?	Dll4/Notch	pro-angiogenesis	↑	(Feng et al. 2015)
NUMBL	NF-κB	pro-inflammatory	↑	((Zhai et al. 2016); (Vaira et al. 2013))
IKBKE	NLRP3	pro-inflammatory	↑	((Robson et al. 2012); (Patel et al. 2015))
SOCS-2	M1 macrophage polarization	pro-inflammatory	↑	((Spence et al. 2013); (Lee et al. 2016))
Scrib	M1 macrophage polarization	pro-inflammatory	↑	((Zheng et al. 2016); (Vaira et al. 2012))
Scrib	ROS	pro-inflammatory	↑	((Zheng et al. 2016); (Vaira et al. 2012))
ICAM-1	directly	anti-adhesion	↓	(Liu et al. 2013)
CX3CR1	directly	anti-adhesion	↓	((Wouters et al. 2017); (Luo and Lin 2016))
MDR1	directly	intestinal cholesterol reabsorption	↑	((Tous et al. 2005); (Hong et al. 2010))
WNK4	directly	hypertension	↓	((Takahashi et al. 2017); (Mao et al. 2010); (Li et al. 2011))
p21^WAF1^	directly	proliferation	↑	((Khanna 2009); (Yang et al. 1996); (Yoon et al. 2011))
p53	p21^WAF1^	proliferation	↑	((Guevara et al. 1999); (Yoon et al. 2011))
p27	directly	proliferation	↑	((Hong et al. 2010); (Diez-Juan and Andres 2001))
PTEN	directly	proliferation	↑	((Yuan et al. 2012); (Li et al. 2009); (Chen et al. 2004); (Huang and Kontos 2002); (Kakizaki et al. 2017))
NGFR	directly	anti-apoptosis	↑	((Chaldakov et al. 2001); (Wang et al. 2000); (Lee et al. 2016))
Caspase-8	directly	anti-apoptosis	↑	(Lee et al. 2016)

↑, stimulatory effect; ↓, inhibitory effect; As, atherosclerosis

## MiR-296 in angiogenesis

Neovascularization in atherosclerotic lesions plays an important role in plaque growth and instability (Camaré et al. 2017). MiR-296 belongs to the family of “angiomirs” due to its functions associated with angiogenesis (Würdinger et al. 2008). MiR-296 and angiogenesis were significantly upregulated following cerebral ischemic injury (Feng et al. 2015). The formation of capillary-like structures of human umbilical vein endothelial cells (HUVEC) was markedly enhanced by adenovirus-mediated overexpression of miR-296 (Feng et al. 2015). The sequence-specific inhibition of miR-296 by intravenous injection of cholesterol-conjugated antagomir can reduce neovascularization in a mouse tumor model (Würdinger et al. 2008).

The vascular endothelial growth factor (VEGF) and Notch signaling pathways are crucial in angiogenesis that is promoted by VEGF and inhibited by Notch under most developmental or pathological conditions (Kim et al. 2017; Mao and Meng 2017). Overexpression of miR-296 was observed to increase the expression levels of VEGF and vascular endothelial growth factor receptor 2 (VEGFR2) and simultaneously reduce the expression levels of delta-like ligand 4 (DLL4) and Notch1 (Feng et al. 2015). One of the miR-296 targets is hepatocyte growth factor-regulated tyrosine kinase substrate (HGS). MiR-296 can reduce the levels of HGS and HGS-mediated degradation of VEGFR2 and platelet-derived growth factor receptor-β (PDGFR-β) (Würdinger et al. 2008). VEGFR and PDGFR are the targets of anti-angiogenic therapies (Batchelor et al. 2007; Shih and Holland 2006). The Notch pathway lies downstream of the VEGF signaling, and the activation of VEGF signaling increases the expression of Notch ligands DLL4 (Liu et al. 2014; Lobov et al. 2007). This finding is contradictory to the research showing that miR-296 promotes VEGF signaling and inhibits the Notch pathway. It is possible that there is another target gene for miR-296, which downregulates the expression of DLL4 and Notch. Further research is needed to elucidate the mechanisms by which miR-296 regulates the angiogenesis.

## MiR-296 in inflammatory response

### MiR-296 regulates NF-кB pathway

In miR-296 overexpressed HCT116 cells, western blot analysis showed that nuclear transcription factor-κB (NF-κB) P65 protein was decreased, while the pP65 level was elevated with enhanced nuclear import. On the contrary, NF-κB P65 level was decreased in cells with low expression of miR-296 (Zhai et al. 2016). Therefore, these findings suggest that miR-296 plays an important role in regulating NF-κB signaling pathway.

In mammals, there is a Numb homolog called “Numb-like” or NumbL, that is a cytoplasm protein and has redundant functions in embryonic neurogenesis. The studies have shown that overexpression of NumbL can inhibit NF-κB activation induced by TNFα and IL-1β, and knockdown of NumbL promotes NF-κB activation, (Ma et al. 2008) suggesting that NumbL plays a negative effect on NF-κB activation. Transfection of A549 NSCLC (non-small cell lung cancer) cells with miR-296-5p was found to inhibit the mRNA and protein expression of NumbL. Two hypothetical miR-296-5p -responsive sites were predicted in the NumbL 3′UTR. The combined mutagenesis of these two sites reversed miR-296-5p repression of NumbL regulatory sequences, suggesting that NumbL is a direct gene target of miR-296-5p (Vaira et al. 2013). Taken together, the current findings suggest that miR-296 may play an inflammatory role by targeted inhibition of NumbL and subsequent NF-κB activation.

### MiR-296 regulates IKBKE expression

The inhibitor of κB kinase epsilon (IKBKE, IKKε, or IKKi) has been identified as an emerging modulator that limits chronic inflammation during metabolic disease and atherosclerosis. IKBKE is necessary to limit both the magnitude and temporal dynamics of Nod-like receptor protein 3 (NLRP3) inflammasome priming in macrophages. It has been reported that Ikbke^−/−^ macrophages can secrete a large amount of IL-1β. Furthermore, IKBKE ablation in apoE^−/−^ mice enhances NLRP3 inflammasome priming and promotes atherosclerosis (Patel et al. 2015). MiR-296-5p may play a negative role in the regulation of IKBKE by targeted inhibition. Robson et al. found that miR-296-5p transfection represses luciferase expression of the PGL3-IKBKE luciferase vector, whereas anti-miR-296-5p transfection activates luciferase expression of the PGL3-IKBKE luciferase vector (Robson et al. 2012). In addition, it was reported that miR-296-5p does not have any remarkable effect on pGL3-IKBKE-3′UTR-mut construct with a mutated target site (Robson et al. 2012.) Thus, it is possible that miR-296-5p may promote inflammation and atherosclerosis through directly targeting IKBKE.

### MiR-296 regulates macrophage polarization

It has been widely acknowledged that M1-polarized macrophages play a pro-inflammatory role in atherosclerosis, whereas M2-polarized macrophages possess an anti-inflammatory effect (De Paoli et al. 2014; Tabas and Bornfeldt 2016). Suppressors of cytokine signaling (SOCS) may be a candidate for controlling leukocyte differentiation and helping shape inflammatory reactions. Recent studies have shown that SOCS facilitates macrophage development and polarization by regulating cytokine expression. SOCS-2 can direct the polarization toward M2 macrophages, and the absence of SOCS-2 resulted in increased polarization toward M1 macrophages (Spence et al. 2013). Overexpression of miR-296-3p decreased the expression of SOCS-2. The underlying mechanism may be that miR-296-3p inhibits the expression of STAT5A, which reportedly activates the expression of SOCS-2 at the transcriptional level (Lee et al. 2016). Therefore, miR-296-3p may regulate macrophage polarization toward M1 macrophages by downregulating the STAT5A-SOCS-2 pathway.

The polarity protein Scribble (Scrib) is involved in infection and inflammation. Downregulation of Scrib expression significantly impaired phorbol 12-myristate 13-acetate (PMA)-induced generation of reactive oxygen species (ROS) in macrophages. Furthermore, the ablation of Scrib promotes M1 macrophage polarization and inflammation (Zheng et al. 2016). Interestingly, the absence of miR-296 causes an aberrant increase in and mislocalization of Scrib in human tumors, leading to exaggerated random cell migration, and tumor cell invasion (Vaira et al. 2012). Further studies have demonstrated that miR-296 can significantly suppress Scrib protein expression by inhibiting Scrib 3′UTR activity, in which Scrib was validated as a target gene directly suppressed by miR-296. These results suggest that miR-296 promotes macrophage polarization toward M1 macrophages probably by repressing Scrib expression.

### MiR-296 regulates adhesion molecules

Adhesion molecules play an important role in mediating the adhesion of leukocytes and monocytes to endothelial cells, which can accelerate arteriosclerosis development. Intercellular adhesion molecule-1 (ICAM-1) is considered as one of the important risk factors involved in atherosclerosis progression. Liu et al. observed that exogenous expression of miR-296-3p significantly inhibits the luciferase activity of the wild-type 3′UTR of the human ICAM-1 reporter, but does not affect the luciferase activity of the mutant reporter. Furthermore, ICAM-1 protein is downregulated in P69-miR-296-3p cell lines and is reversely upregulated in M12-antimiR-296-3p cell lines, as analyzed by both flow cytometry and immunoblotting (Liu et al. 2013). These results demonstrate that miR-296-3p inhibits the expression of ICAM-1 and may be involved in the regression of atherosclerosis.

CX3C chemokine receptor 1 (CX3CR1) has been identified as an important adhesion molecule in migration, adhesion and recruitment of monocytes during the pathogenesis of atherosclerosis (Wouters et al. 2017) CX3CR1 was validated as one of the direct target genes of miR-296-3p. Dual reporter assays have shown that introduction of miR-296-3p in A549 cells inhibits the activity of a luciferase reporter fused to the wild-type 3′UTR of CX3CR1, but does not inhibit that of a reporter fused to a mutant version of the 3′UTR. The introduction of miR-296-3p in A549 and H157 cells reduces CX3CR1 expression at the mRNA and protein levels (Luo and Lin 2016). These results suggest that miR-296-3p may inhibit monocyte adhesion through targeting CX3CR1.

## MiR-296 in cholesterol metabolism

Multidrug resistance protein 1 (MDR1), also known as ATP-binding cassette sub-family B member 1 (ABCB1) or P-glycoprotein 1, is an important protein of cell membrane that functions as a regulator of cholesterol metabolism. In vitro *and* in vivo studies have shown that MDR1 could redistribute cholesterol from the inner leaflet of the membrane to the outer leaflet (Garrigues et al. 2002) or from the membrane to the endoplasmic reticulum to enhance cholesterol esterification (Batetta et al. 2001). Furthermore, MDR1 could be involved in the intestinal cholesterol reabsorption and the susceptibility to atherosclerosis (Tous et al. 2005). The studies have shown that downregulation of miR-296 results in a significant decrease in MDR1 expression. In addition, cotransfection of the MDR1 reporter gene with an increasing amount of antagomirs of miR-296 results in a substantially linear decrease in MDR1 promoter activity, indicating that MDR1 may be a target gene for miR-296 (Hong et al. 2010). However, the mechanism for miR-296 to up-regulate MDR1 is not clear. It was assumed that the effects of miR-296 might depend on not only the promoter sequences but also the association of miR-296 with different cofactors. These studies suggest miR-296 may regulate cholesterol metabolism and promote the formation of atherosclerotic lesions.

## MiR-296 in hypertension

With-no-lysine kinase 4 (WNK4) regulates electrolyte homeostasis and blood pressure. WNK4 is a hypertension causing gene involved in the regulation of salt-sensitive hypertension (Takahashi et al. 2017). Human WNK4 (hWNK4) 3′UTR acts as the enhancer through distant crosstalk with the hWNK4 promoter in a cell-specific manner. Meanwhile, the hWNK4 3′UTR contains a miR-296 binding site as predicted by bioinformatics algorithms and confirmed by a reporter assay. MiR-296 was able to downregulate the expression of hWNK4 at the posttranscriptional level in cell-specific pattern as shown by real-time quantitative PCR and western blot assays (Mao et al. 2010). These data demonstrate that miR-296 may regulate blood pressure by suppressing hWNK4 expression through targeting on its 3′UTR. In addition, Li et al. analyzed that the expression levels of some selected miRNAs, including miR-296-5p, in circulating endothelial cells and endothelial progenitor cells and found that the expression of miR-296-5p is lower in hypertensive patients than healthy control subjects, (Li et al. 2011) suggesting its potential role in the regulation of blood pressure.

## MiR-296 in cellular proliferation and apoptosis

### MiR-296 regulates cell proliferation

It has been shown that overexpression of miR-296-5p significantly promotes gastric cancer growth, (Li et al. 2014) suggesting that miR-296-5p may have a role in cell proliferation. It is known that induction of endogenous p53, (Guevara et al. 1999) p21^WAF1^ (Khanna 2009; Yang et al. 1996) or p27(Diez-Juan and Andres 2001) limits the hyperplastic growth of vascular smooth muscle cells (VSMCs) and macrophages and protects against diet-induced atherosclerosis. In addition, p53/p21 axis plays a central role in the DNA damage response and cell cycle control (Pelletier et al. 2012). Yoon and his colleagues reported that synthetic pre-miR-296-transfected cells show the decreased levels of p53 and p21^WAF1^ transcripts, and anti-miR-296 increases p53 and p21^WAF1^ transcript levels. By monitoring the activities of a luciferase reporter connected to p53 and p21^WAF1^ untranslated regions, those authors demonstrated that miR-296 has a weak but significant effect on the luciferase-p53–3′UTR, and it interacts with the p21^WAF1^ HU region in 3′UTR and decreases p21^WAF1^ mRNA level (Yoon et al. 2011). MiR-296 also regulates the expression of p27. In miR-296 antagomir-treated cells, both the protein and mRNA levels of p27 were decreased, suggesting both transcriptional and translational regulation of p27 by miR-296 (Hong et al. 2010). miR-296 may promote proliferation of VSMCs and macrophages through inhibition of p53, p21^WAF1^ and p27.

The phosphatase and tensin homologue (PTEN) gene is protective in the progression of atherosclerosis (Li et al. 2009; Yuan et al. 2012). Overexpression of PTEN was found to inhibit PDGF-induced phosphorylation of protein kinase B (Akt), p70-S6 kinase, glycogen synthase kinase 3 (GSK3), and thus results in decreased cell proliferation and migration in VSMCs (Chen et al. 2004; Huang and Kontos 2002; Yuan et al. 2012). More recently, Kakizaki and his colleagues found that PTEN is a direct target of miR-296-3p (Kakizaki et al. 2017). The 3′UTR of PTEN harbors a putative miRNA binding site, which has been shown to specifically bind to miR-296-3p. Following transfection with the miR-296-3p, the protein levels of PTEN was markedly decreased. Further analysis showed that miR-296-3p can regulate the phosphoinositide 3-kinase (PI3K)/Akt signaling pathway by targeting PTEN (Kakizaki et al. 2017). These studies suggest that miR-296-3p has an important role in regulating proliferation and migration of VSMCs by directly targeting the PTEN 3′UTR for repression.

### MiR-296 regulates apoptosis

MiR-296 may also suppress apoptosis through nerve growth factor receptor (NGFR) and caspase-8. NGFR, also known as p75NTR, is a neurotrophin receptor that is involved in the pathogenesis of arterial diseases, which is highly expressed in the atherosclerotic adventitia (Chaldakov et al. 2001). NGFR is expressed exclusively in neointimal VSMCs, and the binding of the neurotrophins to NGFR induces cellular apoptosis (Wang et al. 2000). Lee et al. found that the 3′UTR of NGFR harbors a putative miRNA binding site, which specifically binds to miR-296-5p as validated using a luciferase reporter assay. Moreover, the protein and mRNA expression of NGFR is downregulated by miR-296-5p, suggesting that NGFR may be a novel target of miR-296-5p. (Lee et al. 2016). Furthermore, caspase-8, which plays an obligatory role in apoptosis initiation, is also a target of miR-296-5p. MiR-296-5p can directly bind to the 3′UTR of caspase-8 and downregulate its protein and mRNA expression (Lee et al. 2016). In addition, upregulation of miR-296-3p could reduce the resistance of αTC1–6 cells to apoptosis induced by cytokines, include IFN-γ, IL-1β and TNF-α. MiR-298-5p, which is located in the same chromosome locus, as miR-296-3p, also has similar effects on apoptosis (Barbagallo et al. 2013).

## Conclusions

**Fig. 2:**
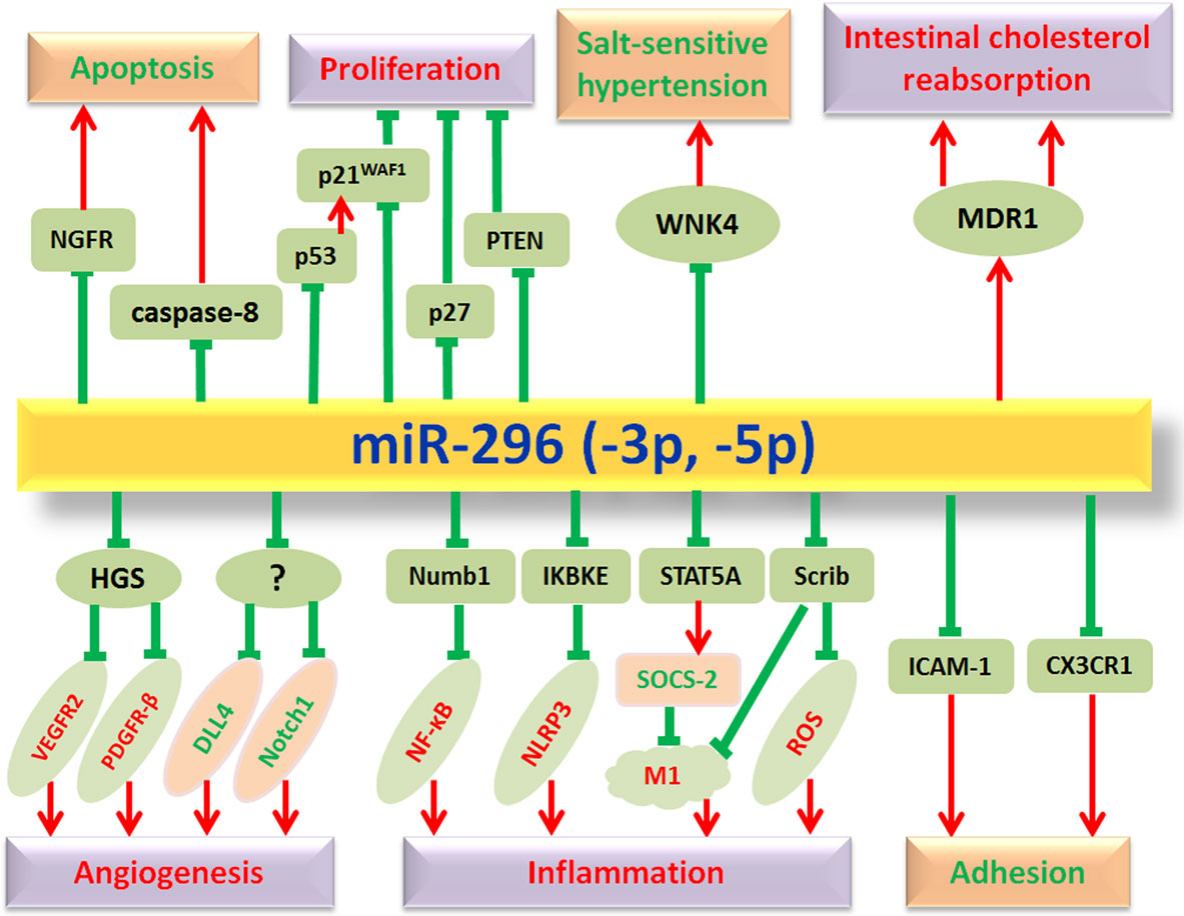
The roles of miR-296 and their target genes in atherosclerosis. MiR-296 promotes angiogenesis via reducing the levels of HGS and then elevating VEGFR2 and PDGFR-β, and simultaneously reducing the expression levels of DLL4 and Notch1 (the mechanism is not clear). MiR-296 promotes the inflammatory response. MiR-296 targeted inhibition of NumbL and subsequent NF-κB activation; miR-296 represses IKBKE and then enhances NLRP3 inflammasome; miR-296 inhibits the expression of STAT5A and then represses SOCS-2 and promotes M1 macrophage polarization; miR-296 directly suppresses Scrib and subsequently promotes M1 macrophage polarization and ROS generation, these entire can promote inflammation. MiR-296 could repress ICAM-1 and CX3CR1 and then inhibit monocyte adhesion. MiR-296 promotes MDR1 expression and subsequent intestinal cholesterol reabsorption. MiR-296 inhibits WNK4 expression and downregulates salt-sensitive hypertension. MiR-296 suppresses p53, p21WAF1, p27 and PTEN expression and then promotes cells proliferation. MiR-296 targeted inhibition of NGFR and caspase-8 promotes cell apoptosis. The black word: Direct target of miR-296, the red word: Expression increased, the green word: Expression decreased. : promote, : inhibit

MiR-296 can play a critical role in the initiation and progression of atherosclerosis through regulating its target gene expression. These target genes of miR-296 are involved in the modulation of angiogenesis, inflammatory response, cholesterol metabolism, hypertension, cellular proliferation and apoptosis (Fig. 2).

The immunomodulatory role of miR-296 appears complicated. The role of miR-296 as potent regulators of angiogenic phenotype has been widely accepted. Overexpression of miR-296 can increase the expression levels of VEGF, and simultaneously inhibit the Notch pathway (Feng et al. 2015). It does not conform to the fact that activation of VEGF signaling increases the expression of Notch ligand DLL4, suggesting that miR-296 may regulate multiple target genes to promote angiogenesis. However, miR-296-3p has the potential to inhibit atherosclerosis by regulating adhesion molecules, including ICAM-1 and CX3CR1 (Liu et al. 2013; Luo and Lin 2016). Furthermore, miR-296 also exhibits an anti- hypertensive effect by suppressing hWNK4 expression, (Mao et al. 2010) because there is a low expression of miR-296-5p in hypertensive patients compared with healthy control subjects (Li et al. 2011). In addition, miR-296-3p can reduce the resistance of αTC1–6 cells to apoptosis induced by cytokines, such as IFN-γ, IL-1β and TNF-α, suggesting its involvement in the regulation of Type 2 diabetes (Barbagallo et al. 2013; Dunning and Gerich 2007). Taken together, increasing evidence supports the hypothesis that miR-296 may be served as a potential indicator of atherosclerosis.

## Future perspectives and challenges

In the future, ongoing studies of miR-296 will be necessary to clarify its role in atherosclerosis and animal models of various diseases. There are some significant and insightful questions that need to be answered: 1) what regulates miR-296 levels in the biological process of atherosclerosis? 2) How does a single miR-296 strictly regulate the expression of multi-target genes in vivo and in vitro? 3) Are there more miR-296 target genes that can affect the development and progression of atherosclerosis? 4) Does miR-296 influence the progress of atherosclerosis by targeting different signaling pathways in different cell types and tissues? Finally, it remains to be investigated whether miR-296 can be served as a diagnostic and prognostic marker for atherosclerosis based on the identified roles. Given that the functions of miR-296, in particular, its roles in atherosclerosis, have not been researched extensively, understanding the molecular mechanisms and cellular pathways controlled by miR-296 will certainly promote promoting miR-296 research in atherosclerosis.

## References

[CR1] Andreou I, Sun X, Stone PH (2015). miRNAs in atherosclerotic plaque initiation, progression, and rupture[J]. Trends Mol Med.

[CR2] Barbagallo D, Piro S, Condorelli AG (2013). miR-296-3p, miR-298-5p and their downstream networks are causally involved in the higher resistance of mammalian pancreatic alpha cells to cytokine-induced apoptosis as compared to beta cells[J]. BMC Genomics.

[CR3] Barquera S, Pedroza-Tobias A, Medina C (2015). Global overview of the epidemiology of atherosclerotic cardiovascular disease[J]. Arch Med Res.

[CR4] Batchelor TT, Sorensen AG, di Tomaso E (2007). AZD2171, a pan-VEGF receptor tyrosine kinase inhibitor, normalizes tumor vasculature and alleviates edema in glioblastoma patients[J]. Cancer Cell.

[CR5] Batetta B, Mulas MF, Petruzzo P (2001). Opposite pattern of MDR1 and caveolin-1 gene expression in human atherosclerotic lesions and proliferating human smooth muscle cells[J]. Cell Mol Life Sci.

[CR6] Buckley ML, Ramji DP (2015). The influence of dysfunctional signaling and lipid homeostasis in mediating the inflammatory responses during atherosclerosis[J]. Biochim Biophys Acta.

[CR7] Camaré C, Pucelle M, Nègre-Salvayre A (2017). Angiogenesis in the atherosclerotic plaque[J]. Redox Biol.

[CR8] Chaldakov GN, Stankulov IS, Fiore M (2001). Nerve growth factor levels and mast cell distribution in human coronary atherosclerosis[J]. Atherosclerosis.

[CR9] Chen WJ, Lin KH, Lai YJ (2004). Protective effect of propylthiouracil independent of its hypothyroid effect on atherogenesis in cholesterol-fed rabbits: PTEN induction and inhibition of vascular smooth muscle cell proliferation and migration[J]. Circulation.

[CR10] De Paoli F, Staels B, Chinetti-Gbaguidi G (2014). Macrophage phenotypes and their modulation in atherosclerosis[J]. Circ J.

[CR11] Diez-Juan A, Andres V (2001). The growth suppressor p27(Kip1) protects against diet-induced atherosclerosis[J]. FASEB J.

[CR12] Dunning BE, Gerich JE (2007). The role of alpha-cell dysregulation in fasting and postprandial hyperglycemia in type 2 diabetes and therapeutic implications[J]. Endocr Rev.

[CR13] Ebert MS, Sharp PA (2012). Roles for microRNAs in conferring robustness to biological processes[J]. Cell.

[CR14] Fang L, Ellims AH, Moore XL (2015). Circulating microRNAs as biomarkers for diffuse myocardial fibrosis in patients with hypertrophic cardiomyopathy[J]. J Transl Med.

[CR15] Feng J, Huang T, Huang Q (2015). Proangiogenic microRNA296 upregulates vascular endothelial growth factor and downregulates Notch1 following cerebral ischemic injury[J]. Mol Med Rep.

[CR16] Garrigues A, Escargueil AE, Orlowski S (2002). The multidrug transporter, P-glycoprotein, actively mediates cholesterol redistribution in the cell membrane[J]. Proc Natl Acad Sci U S A.

[CR17] Giral H, Kratzer A, Landmesser U (2016). MicroRNAs in lipid metabolism and atherosclerosis[J]. Best Pract Res Clin Endocrinol Metab.

[CR18] Guevara NV, Kim HS, Antonova EI (1999). The absence of p53 accelerates atherosclerosis by increasing cell proliferation in vivo[J]. Nat Med.

[CR19] Hong L, Han Y, Zhang H (2010). The prognostic and chemotherapeutic value of miR-296 in esophageal squamous cell carcinoma[J]. Ann Surg.

[CR20] Huaman MA, Henson D, Ticona E, et al. Tuberculosis and cardiovascular disease: linking the epidemics[J]. Trop Dis Travel Med Vaccines. 2015;1 10.1186/s40794-015-0014-5 PMC4729377 26835156

[CR21] Huang J, Kontos CD (2002). Inhibition of vascular smooth muscle cell proliferation, migration, and survival by the tumor suppressor protein PTEN[J]. Arterioscler Thromb Vasc Biol.

[CR22] Inoue M, Itoh H, Ueda M (1998). Vascular endothelial growth factor (VEGF) expression in human coronary atherosclerotic lesions: possible pathophysiological significance of VEGF in progression of atherosclerosis[J]. Circulation.

[CR23] Kakizaki T, Hatakeyama H, Nakamaru Y (2017). Role of microRNA-296-3p in the malignant transformation of sinonasal inverted papilloma[J]. Oncol Lett.

[CR24] Kanter JE, Averill MM, Leboeuf RC (2008). Diabetes-accelerated atherosclerosis and inflammation[J]. Circ Res.

[CR25] Khanna AK (2009). Enhanced susceptibility of cyclin kinase inhibitor p21 knockout mice to high fat diet induced atherosclerosis[J]. J Biomed Sci.

[CR26] Kim YM, Kim SJ, Tatsunami R (2017). ROS-induced ROS release orchestrated by Nox4, Nox2, and mitochondria in VEGF signaling and angiogenesis[J]. Am J Physiol Cell Physiol.

[CR27] Laffont B, Rayner KJ (2017). MicroRNAs in the pathobiology and therapy of atherosclerosis[J]. Can J Cardiol.

[CR28] Lee H, Hwang SJ, Kim HR (2016). Neurofibromatosis 2 (NF2) controls the invasiveness of glioblastoma through YAP-dependent expression of CYR61/CCN1 and miR-296-3p[J]. Biochim Biophys Acta.

[CR29] Lee H, Shin CH, Kim HR (2016). MicroRNA-296-5p promotes invasiveness through Downregulation of nerve growth factor receptor and Caspase-8[J]. Mol Cells.

[CR30] Li K, Yao W, Zheng X (2009). Berberine promotes the development of atherosclerosis and foam cell formation by inducing scavenger receptor a expression in macrophage[J]. Cell Res.

[CR31] Li S, Zhu J, Zhang W (2011). Signature microRNA expression profile of essential hypertension and its novel link to human cytomegalovirus infection[J]. Circulation.

[CR32] Li T, Lu YY, Zhao XD (2014). MicroRNA-296-5p increases proliferation in gastric cancer through repression of caudal-related homeobox 1[J]. Oncogene.

[CR33] Liu X, Chen Q, Yan J (2013). MiRNA-296-3p-ICAM-1 axis promotes metastasis of prostate cancer by possible enhancing survival of natural killer cell-resistant circulating tumour cells[J]. Cell Death Dis.

[CR34] Liu Z, Fan F, Wang A (2014). Dll4-notch signaling in regulation of tumor angiogenesis[J]. J Cancer Res Clin Oncol.

[CR35] Lobov IB, Renard RA, Papadopoulos N (2007). Delta-like ligand 4 (Dll4) is induced by VEGF as a negative regulator of angiogenic sprouting[J]. Proc Natl Acad Sci U S A.

[CR36] Luo W, Lin Y (2016). Meng S, et al. miRNA-296-3p modulates chemosensitivity of lung cancer cells by targeting CX3CR1[J]. Am J Transl Res.

[CR37] Ma Q, Zhou L, Shi H (2008). NUMBL interacts with TAB2 and inhibits TNFalpha and IL-1beta-induced NF-kappaB activation[J]. Cell Signal.

[CR38] Mao J, Li C, Zhang Y (2010). Human with-no-lysine kinase-4 3'-UTR acting as the enhancer and being targeted by miR-296[J]. Int J Biochem Cell Biol.

[CR39] Mao R, Meng S (2017). Gu Q, et al. AIBP limits angiogenesis through gamma-Secretase-mediated Upregulation of notch signaling[J]. Circ Res.

[CR40] Meng QL, Liu F, Yang XY (2014). Identification of latent tuberculosis infection-related microRNAs in human U937 macrophages expressing Mycobacterium tuberculosis Hsp16.3[J]. BMC Microbiol.

[CR41] Patel MN, Bernard WG, Milev NB (2015). Hematopoietic IKBKE limits the chronicity of inflammasome priming and metaflammation[J]. Proc Natl Acad Sci U S A.

[CR42] Pelletier J, Dayan F, Durivault J (2012). The asparaginyl hydroxylase factor-inhibiting HIF is essential for tumor growth through suppression of the p53-p21 axis[J]. Oncogene.

[CR43] Reddy MA, Das S, Zhuo C (2016). Regulation of vascular smooth muscle cell dysfunction under diabetic conditions by miR-504[J]. Arterioscler Thromb Vasc Biol.

[CR44] Robson JE, Eaton SA, Underhill P (2012). MicroRNAs 296 and 298 are imprinted and part of the GNAS/Gnas cluster and miR-296 targets IKBKE and Tmed9[J]. RNA.

[CR45] Selbach M, Schwanhausser B, Thierfelder N (2008). Widespread changes in protein synthesis induced by microRNAs[J]. Nature.

[CR46] Shih AH, Holland EC (2006). Platelet-derived growth factor (PDGF) and glial tumorigenesis[J]. Cancer Lett.

[CR47] Spence S, Fitzsimons A, Boyd CR (2013). Suppressors of cytokine signaling 2 and 3 diametrically control macrophage polarization[J]. Immunity.

[CR48] Tabas I, Bornfeldt KE (2016). Macrophage phenotype and function in different stages of atherosclerosis[J]. Circ Res.

[CR49] Takahashi D, Mori T, Sohara E (2017). WNK4 is an Adipogenic factor and its deletion reduces diet-induced obesity in mice[J]. EBioMedicine.

[CR50] Tous M, Ribas V, Ferre N (2005). Turpentine-induced inflammation reduces the hepatic expression of the multiple drug resistance gene, the plasma cholesterol concentration and the development of atherosclerosis in apolipoprotein E deficient mice[J]. Biochim Biophys Acta.

[CR51] Vaira V, Faversani A, Dohi T (2012). miR-296 regulation of a cell polarity-cell plasticity module controls tumor progression[J]. Oncogene.

[CR52] Vaira V, Faversani A, Martin NM (2013). REGULATION OF LUNG CANCER METASTASIS BY Klf4-Numb-like SIGNALING[J]. Cancer Res.

[CR53] Wang S, Bray P, Mccaffrey T (2000). P75(NTR) mediates neurotrophin-induced apoptosis of vascular smooth muscle cells[J]. Am J Pathol.

[CR54] Wei Y, Nazari-Jahantigh M, Chan L (2013). The microRNA-342-5p fosters inflammatory macrophage activation through an Akt1- and microRNA-155-dependent pathway during atherosclerosis[J]. Circulation.

[CR55] Wouters K, Gaens K, Bijnen M (2017). Circulating classical monocytes are associated with CD11c+ macrophages in human visceral adipose tissue[J]. Sci Rep.

[CR56] Würdinger T, Tannous BA, Saydam O (2008). miR-296 regulates growth factor receptor overexpression in angiogenic endothelial cells[J]. Cancer Cell.

[CR57] Yang ZY, Simari RD, Perkins ND (1996). Role of the p21 cyclin-dependent kinase inhibitor in limiting intimal cell proliferation in response to arterial injury[J]. Proc Natl Acad Sci U S A.

[CR58] Yoon AR, Gao R, Kaul Z (2011). MicroRNA-296 is enriched in cancer cells and downregulates p21WAF1 mRNA expression via interaction with its 3′ untranslated region[J]. Nucleic Acids Res.

[CR59] Yuan M, Wang X, Zhan Q (2012). Association of PTEN genetic polymorphisms with atherosclerotic cerebral infarction in the Han Chinese population[J]. J Clin Neurosci.

[CR60] Zhai H, Sui M, Jiang L (2016). MiR-296 promotes colorectal cancer cells growth through regulating NF-κB[J]. Int J Clin Exp Pathol.

[CR61] Zheng L, Leung E, Lee N (2015). Differential MicroRNA expression in human macrophages with Mycobacterium tuberculosis infection of Beijing/W and non-Beijing/W strain types[J]. PLoS One.

[CR62] Zheng W, Umitsu M, Jagan I (2016). An interaction between scribble and the NADPH oxidase complex controls M1 macrophage polarization and function[J]. Nat Cell Biol.

